# Identification of three dietary groups in French university students and their associations with nutritional quality and environmental impact

**DOI:** 10.3389/fnut.2023.1323648

**Published:** 2023-12-20

**Authors:** Laura Arrazat, Sophie Nicklaus, Blandine de Lauzon-Guillain, Lucile Marty

**Affiliations:** ^1^Centre des Sciences Du Goût et de l’Alimentation, CNRS, INRAE, Institut Agro, Université de Bourgogne, Dijon, France; ^2^Université Paris Cité and Université Sorbonne Paris Nord, Inserm, INRAE, Center for Research in Epidemiology and StatisticS, Paris, France

**Keywords:** diet, nutritional quality, environmental impact, university students, cluster analysis

## Abstract

**Introduction:**

The student period is associated with changes in eating habits, usually leading to diets of lower nutritional quality. However, some variability may exist in students’ dietary patterns. We aimed to describe French students’ diets and identify dietary groups that may vary in nutritional quality and environmental impact.

**Methods:**

A representative sample of French students (N = 582) for age, sex and scholarship status completed an online 125-item food frequency questionnaire. The nutritional quality of diets was assessed by a score of adherence to the French nutritional guidelines (sPNNS-GS2 score, ranging from−17 to 11.5) and its environmental impact by greenhouse gas emissions for an isocaloric diet (GHGE). An ascending hierarchical classification analysis on food and beverage intakes led to three dietary groups. Between-group differences in food consumption, dietary indicators and sociodemographic characteristics were investigated using ANOVA models.

**Results:**

The average sPNNS-GS2 score of students’ diets was −0.8 ± 2.8, representing a 57% coverage of French nutritional recommendations, and GHGE were 5.4 ± 1.7 kg eCO_2_/2000 kcal. The three dietary groups were: a *healthy diet* group (20% of the sample) with the highest nutritional quality and high GHGE, which included older students with a higher level of physical activity; a *Western diet* group (40%) with the worst nutritional quality and high GHGE, which included more students who lived with their parents; and a *frugal diet* group (40%) with the lowest energy intake, intermediate nutritional quality, and low GHGE, which included more students who lived alone.

**Conclusion:**

None of the dietary groups optimized both nutritional quality and environmental impact simultaneously, which suggests an apparent incompatibility in the student population between these two sustainability dimensions. These findings emphasize the need for tailored public health policies that acknowledge the diversity of student eating patterns and address specific individual barriers to healthy and sustainable diets.

## 1 Introduction

Students attending higher education represent 4% of the French population and 44% of young adults (18–25 years old) (1). They form a specific population at a sensitive period between childhood and adulthood (2). Student years constitute a period of identity exploration as individuals draw away from their families (3), usually resulting in the deconstruction of eating habits formed during childhood and the gradual establishment of new eating habits (4–6). Reshaping habits when transitioning to higher education are generally associated with diets of poor nutritional quality (6–9); therefore, this period may be critical for promoting healthy and sustainable dietary habits (10). Unhealthy eating behaviors have been identified as factors contributing to weight gain in the initial year of university and are associated with adverse health outcomes such as coronary artery disease (11, 12). In addition to these health concerns, unhealthy eating patterns also impact sleep and cognitive functions, which are known to be interconnected with academic performance (13–15). Therefore, there is a growing body of research that shows an association between a more favorable dietary intake and higher levels of academic achievement, underlining the importance of healthy eating in this population (16–18). Finally, in a context where food choices are increasingly linked to both population and planet health (19, 20), the current level of nutritional quality and the environmental impact of students’ diets seem important to assess in order to identify how to improve both aspects.

Although several studies have demonstrated that diets combining good nutritional quality and a low environmental impact are achievable (19, 21, 22), maximizing both might not be easy for students. For instance, the high consumption of sweet, salty, and fatty foods and sweetened drinks observed in this population is related to lower nutritional quality but also a lower environmental impact (7, 9). Observational studies, including nutritional quality and environmental impact indicators, are lacking in student populations. To our knowledge, these two dimensions of sustainability have been analyzed together only in a sample of Spanish students, where a diet with a higher nutritional quality score was associated with a slightly higher environmental impact (23).

Although the dietary patterns of students have been described as quite homogeneous, particularly regarding their poor nutritional quality (7), some variability has also been identified among this population (24–26). A study conducted on 1,448 United Kingdom students proposed four major dietary patterns: a ‘vegetarian’ pattern, a ‘snacking’ pattern, a ‘health-conscious’ pattern and a ‘convenience, red meat & alcohol’ pattern (24). As a step further, dietary patterns in the student population beyond food group consumption and in terms of nutritional quality and environmental impact would be interesting to describe. Finally, as diverse dietary groups may exist in this population, identifying their sociodemographic characteristics or their living situations is important, which may allow the identification of subgroups of students with specific issues that may impede reaching adequate nutritional quality and/or an acceptable environmental impact.

In France, many studies aiming to characterize the diet quality and environmental impact of the general population have been carried out (21, 27, 28), but to our knowledge, no study has investigated French higher education students’ diets. Data on this topic are needed to inform public policies on how to initiate transitions toward healthy and sustainable diets in this population. The first aim of the present study was to describe the overall dietary characteristics of a representative sample of French students. The second aim was to investigate the variability in dietary patterns among French students and describe these dietary groups in terms of nutritional quality, environmental impact, and sociodemographic and lifestyle characteristics.

## 2 Materials and methods

### 2.1 Study design

In this cross-sectional study, participants completed a 30-min online survey on the Qualtrics platform^1^ including three parts: (i) a semi-quantitative food frequency questionnaire (FFQ) assessing dietary intake during the last month, (ii) a series of validated questionnaires to measure various determinants of eating practices (this second part of the questionnaire was not analyzed in the present study), and (iii) questions on sociodemographic characteristics and lifestyle. Participants were informed that the purpose of the study was to investigate their eating habits and gave their consent to participate before starting the survey. Three attention-check questions (e.g., ‘How many times do you eat cars?’) were included in the questionnaire.

### 2.2 Subjects and recruitment

Eligible participants for this study were students enrolled in higher education in Dijon (France), aged over 18 years, fluent in French, and without children. We excluded students who were parents, as the presence of a child can significantly influence dietary habits (29, 30). To be as representative as possible of the French student population, we recruited participants with quotas on sex, age and scholarship status. In France, scholarship status is linked to parental incomes and is considered as an indicator of socio-economic status in the student population. Quotas were calculated in comparison to the representation of each category in the French student population in 2019 (31). Therefore, we stratified our sample by sex (56% female, 44% male), age (63% between 18 and 21 years of age, 26% between 22 and 25 years of age, and 11% over 25 years of age), and scholarship status (37% with a scholarship, indicating a lower socioeconomic status). We aimed to recruit a minimum of 60 participants for each quota-based group, as we hypothesized that the three quota variables (sex, age and scholarship status) would play important roles in shaping dietary practices (7). The group representing the smallest percentage of the total population was the “over 25-year-old” age group (11%). Considering that 60 participants would represent 11% of the total population, a total of 546 participants were required. Thus, we aimed to recruit 600 participants to allow for approximately 10% of potential *a posteriori* exclusions due to poor quality responses based on a previous study (32).

Three recruitment methods were used: social media and more specifically Facebook posts on the Dijon university page, emails from certain universities that agreed to transfer our survey and flyers handed out on the university campus of Dijon. Direct access to the online survey was provided via a link on posts and emails or a QR code printed on flyers. At survey initiation, questions about age, sex, and scholarship status aimed to assess eligibility and completion of quotas before the participants could proceed with the survey. The participants who completed the survey received a 10€ voucher by email. Data were collected between the 1st and 15th of April 2022.

### 2.3 Ethical aspects

This study was conducted in accordance with the Declaration of Helsinki, and the protocol for this study was approved by the ethical evaluation committee for research of Inserm (reference: 22–884, delivered on March 8th, 2022). Written informed consent was obtained from all study participants.

### 2.4 Measures

#### 2.4.1 Participants’ characteristics

Sociodemographic characteristics queried in the survey included age, sex, scholarship status, nationality, current level of education, type of institution, current disciplinary field of study, number of years spent in higher education, parents’ highest level of education, place of residence at the time of the study and other concurrent residents. We also asked the participants to report their weight (kg) and height (cm) to calculate their body mass index (BMI) in kg/m^2^ and whether they were dieting or willing to gain muscle mass. Physical activity level was evaluated using the self-administered International Physical Activity Questionnaire (IPAQ) short-form questionnaire, which allowed us to derive three levels of physical activity: low, moderate and high (33).

#### 2.4.2 Dietary data

Participants declared the frequency and portion sizes of the food items and beverages that they consumed during the month before completing the survey (i.e., March 2022 for all participants) using a validated semi-quantitative FFQ (34). The frequency of consumption of 109 food items, 12 non-alcoholic beverages and 4 alcoholic beverages and 13 spice and herb items was assessed with a 6-item scale ranging from “never” to “several times a day.” Supplement intake was not assessed. Reported frequencies of consumption were transformed into daily frequencies. Portion sizes were estimated by the participants on a 5-level photograph scale adapted from the SU.VI.MAX portion book (35) for 71 food items and 12 non-alcoholic beverages. An average portion size was used for the 38 remaining food items. Alcoholic beverage amounts corresponded to standard serving sizes. Daily intake of each food and beverage item was calculated by multiplying the daily frequency by the estimated portion size. Individual nutrient intakes (g/day) were obtained by multiplying this daily intake by the nutrient content of each item retrieved from the SU.VI.MAX nutrient composition database (36).

The 109 food items, 13 spice and herb items and 16 beverages were grouped into 39 food groups based on the classification used in the 2014–2015 French national dietary survey (Enquête Individuelle Nationale des Consommations Alimentaires, INCA 3) (37). For each food group, we calculated the daily consumption in grams per day and energy-adjusted consumption in grams per 2000 kcal.

We also asked participants to declare the type of diet they were following at the time of the study: omnivorous, flexitarian, pesco-vegetarian, ovo-lacto-vegetarian or vegan.

### 2.5 Diet quality indicators

#### 2.5.1 Nutritional quality

To assess the nutritional quality of the students’ diets, we used a validated indicator for the French population: the simplified PNNS-GS2 score (sPNNS-GS2) (38), which measures adherence to the 2017 French nutritional guidelines. This score attributes positive points to healthy food group consumption (i.e., fruit and vegetables, nuts, legumes, whole-grain food, milk and dairy products, fish, and seafood) and negative points to unhealthy food group consumption (i.e., red meat, processed meat, sugary foods, sweet-tasting beverages, alcoholic beverages, and salt), with a higher score indicating a diet closer to the French nutritional guidelines. As detailed in previous papers that calculated the sPNNS-GS2 based on the same FFQ (28, 32), the original score calculation was slightly modified. As the percentage of energy intake contributed by the added fat could not be recorded with this FFQ, this component was removed from the score calculation. As a consequence, the sPNNS-GS2 score computed for each participant in the present study ranged from −17 to 11.5 instead of −17 to 13.5 (38).

For sensitivity analyzes, we also evaluated the nutritional quality of diets using the PANDiet score, which measures the adequacy of nutrient intake (39, 40).

#### 2.5.2 Environmental impact

To evaluate the environmental impact of students’ diets, we used greenhouse gas emissions (GHGE) in kg eCO_2_/kg derived from the French food environmental impact database Agribalyse 3.0 created by the French Agency for Ecological Transition (ADEME). This database is composed of 2,480 common food items for which GHGE values were calculated using life cycle analyzes (41). The FFQ items were associated with the corresponding food items from Agribalyse 3.0, as described in a previous study (28). To calculate the environmental impact of each participant’s diet, we multiplied the daily intake of each food item by the associated GHGE per kg. We also calculated the energy-adjusted environmental impact of the diets (GHGE per 2000 kcal).

#### 2.5.3 Consumption of organic and locally produced products

The participants were asked how often they consumed organic and locally produced foods for 12 food groups (fruit, vegetables, dairy products, meat and fish, eggs, grains, bread, oil, ready-to-eat meals, biscuits, tea and coffee, and wine and beers) on a 3-point scale: 2 = most of the time, 1 = occasionally and 0 = never. This questionnaire was used in previous studies (28, 42). Organic and local consumption scores were computed as the mean of responses across the 12 food groups (range 0 to 2).

### 2.6 Statistical analyzes

We followed an analytical plan that was registered prior to data collection.^2^ From the 600 participants who completed the survey, we excluded those who failed at least one attention check question and those with extreme energy intake, who were defined as participants in the first and last percentiles of energy intake (p1 = 576 kcal/day and p100 = 7,323 kcal/day).

Dietary groups were identified from food group daily intakes (in g/day) (*N* = 39) using a clustering analysis to group students with similar dietary patterns. The variables used in this analysis were standardized daily food group consumption in grams per day (*N* = 39). Standardization controls for the potential influence of certain food groups with higher weights, such as beverages. A hierarchical ascendant classification was conducted using Ward’s method based on Euclidean distances (proc cluster) and highlighted three distinct clusters composed of 125, 227, and 227 participants. Three deviant participants grouped in a fourth cluster were excluded from the cluster comparison. Although 39 food groups were used in the clustering analysis, for the sake of conciseness when presenting the results, we merged some food groups to obtain 26 simplified food and beverage groups (see the list of food groups, along with corresponding FFQ items, in Supplementary material S1).

For each simplified food group (*N* = 26), intake differences between the three dietary groups were examined using ANOVA and *post hoc* pairwise comparisons with Bonferroni correction for daily and energy-adjusted intakes. Analyzing daily intake enables a comparison of the absolute values used to categorize the three dietary groups–quantitative comparison. However, given the variations in energy intake we observed, we also conducted a comparison of intakes by food group for an isocaloric diet to be able to compare their relative contribution in each dietary group–qualitative comparison. Comparisons were also performed for the following diet quality indicators: macronutrient contents, nutritional quality indicators (sPNNS-GS2, PANDiet), environmental impact (GHGE per day and per 2000 kcal), consumption of organic and locally produced products; as well as for sociodemographic characteristics, level of physical activity and anthropometric data, with ANOVA used for continuous variables and Chi-2 tests used for categorical variables.

Sensitivity analyzes were conducted to investigate whether the characteristics of the dietary groups changed after exclusion of under- and over-reporting participants for energy intake. We used Schofield equations considering weight, height, sex and age to calculate basal metabolic rate. The basal metabolic rate was corrected using the level of physical activity (derived from the results of the International Physical Activity Questionnaire (IPAQ) questionnaire) and compared to the energy intake calculated from the FFQ. Participants were classified as under- (*N* = 158) or over-reporters (*N* = 26) using the cut-off points proposed by Black (43). All the comparison analyzes between the dietary groups described above were replicated after the exclusion of under- and over-reporting participants.

All statistical analyzes were performed using SAS version 9.4 (SAS Institute, Inc. Cary, NC). The level of significance was set at *p* < 0.05 across all preregistered analyzes unless otherwise specified.

## 3 Results

### 3.1 Sample characteristics

Data from 582 participants were included in the analyzes, see the survey flow in Supplementary material S2. The characteristics of these participants are presented in Supplementary material S3. Recruitment quotas were achieved for sex (56% female). We recruited slightly more participants with a scholarship than planned (40% vs. 37%). Regarding age groups, 67% of participants were between 18 and 21 years old (vs. 63% planned), 26% were between 22 and 25 years old (as planned), and 7% were over 25 years of age (vs. 11% planned). Most of our sample consisted of French students (94%) who had been studying for 3.3 years (SD = 2.1). Most of the students lived alone (56%) or with a partner or flatmates (29%).

Regarding anthropometric data, 69% of the participants reported a normal weight status (BMI between 18.5 and 25 kg/m^2^). Some participants declared that they were dieting (9%), and some wanted to gain muscle mass (14%). More than two-thirds of the sample identified themselves as omnivores (67.5%), a quarter as flexitarians (24.6%) and the rest as vegetarian or vegan (7.9%).

### 3.2 Food consumption patterns across dietary groups

Three dietary groups were identified from the hierarchical ascendant classification on daily consumption by food groups representing three distinct dietary patterns: the *healthy diet* group (*N* = 125, 20% of the sample), the *Western diet* group (*N* = 227, 40%) and the *frugal diet* group (*N* = 227, 40%). Table 1 shows the average amounts consumed by food and beverage groups per day and per 2000 kcal in each group and for the whole sample.

**Table 1:** Food and beverage consumptions in grams per day and for an isocaloric diet (/2000 kcal) across the three dietary groups of French university students (*N* = 582).

	Food groups in g/day and beverages in ml/day					Food groups in g/2000 kcal and beverages in ml/2000 kcal				
	Overall	Healthy diet group	Western diet group	Frugal diet group	*p*	Overall	Healthy diet group	Western diet group	Frugal diet group	*p*
	*N* = 582^a^	*N* = 227	*N* = 125	*N* = 227		*N* = 582^a^	*N* = 227	*N* = 125	*N* = 227	
**Food groups**										
Bread and cereals	263 (227)	324 (290)^A^	303 (238)^A^	188 (138)^B^	<0.001	248 (146)	257 (148)^A^	232 (130)^A^	262 (158)^A^	0.068
Dairy products	90.6 (96.4)	114 (134)^A^	103 (89.4)^A^	66.1 (70.0)^B^	<0.001	88.3 (80.5)	91.5 (76.3)^A^	83.6 (67.7)^A^	92.4 (93.5)^A^	0.462
Dairy and soy-based desserts	31.4 (43.0)	27.0 (41.7)^B^	45.3 (50.8)^A^	20.0 (29.5)^B^	<0.001	30.5 (41.5)	24.4 (37.8)^B^	37.5 (47.6)^A^	27.2 (35.9)^A,B^	0.005
Fats and sauces	18.9 (15.9)	16.1 (14.3)^B^	24.6 (18.8)^A^	14.8 (11.4)^B^	<0.001	19.5 (15.0)	15.0 (13.0)^B^	20.6 (15.6)^A^	21.2 (14.9)^A^	<0.001
Egg	45.2 (68.8)	75.8 (105)^A^	40.1 (55.0)^B^	32.8 (47.4)^B^	<0.001	42.0 (52.6)	58.7 (60.4)^A^	30.7 (36.8)^C^	44.2 (58.9)^B^	<0.001
Red meat	36.2 (53.3)	40.0 (64.4)^A^	50.7 (61.1)^A^	19.8 (28.2)^B^	<0.000	31.4 (34.8)	29.6 (36.5)^A,B^	37.6 (36.5)^A^	26.5 (31.1)^B^	0.002
Poultry	30.4 (57.5)	60.8 (107)^A^	27.9 (30.1)^B^	15.4 (17.0)^B^	<0.001	26.3 (34.1)	41.8 (56.8)^A^	22.2 (21.2)^B^	21.8 (23.9)^B^	<0.001
Pork	11.3 (18.9)	8.29 (14.4)^B^	18.9 (24.8)^A^	5.56 (9.42)^B^	<0.001	10.3 (15.8)	6.73 (11.2)^B^	15.1 (19.5)^A^	7.70 (12.5)^B^	<0.001
Processed meat and offal	21.1 (25.1)	16.2 (17.9)^B^	32.3 (32.4)^A^	12.9 (13.6)^B^	<0.001	20.5 (21.7)	13.5 (13.9)^C^	26.1 (24.7)^A^	19.1 (20.8)^B^	<0.001
Fish and seafood	22.5 (36.7)	32.4 (62.0)^A^	27.8 (29.8)^A^	12.1 (16.2)^B^	<0.001	20.5 (27.0)	24.2 (36.4)^A^	21.9 (22.3)^A,B^	17.3 (24.9)^B^	0.043
Vegetables	186 (203)	327 (319)^A^	183 (158)^B^	113 (89.9)^C^	<0.001	190 (182)	305 (275)^A^	155 (138)^B^	163 (124)^B^	<0.001
Pulses	22.4 (51.0)	51.2 (97.5)^A^	15.7 (23.0)^B^	13.3 (18.8)^B^	<0.001	23.8 (50.1)	51.1 (92.7)^A^	13.0 (18.8)^B^	19.8 (28.7)^B^	<0.001
Starchy vegetables	51.9 (51.7)	43.6 (33.8)^B^	73.7 (67.1)^A^	34.5 (30.0)^B^	<0.001	50.8 (44.2)	40.1 (34.6)^B^	59.2 (50.5)^A^	48.6 (40.9)^A,B^	<0.001
Fruits	134 (153)	218 (204)^A^	132 (144)^B^	90.1 (105)^C^	<0.001	131 (137)	186 (159)^A^	109 (125)^B^	125 (129)^B^	<0.001
Sweet products	85.2 (68.2)	72.8 (47.1)^B^	111 (87.2)^A^	67.1 (45.1)^B^	<0.001	83.2 (46.7)	64.6 (37.2)^B^	84.7 (42.8)^A^	92.8 (51.5)^A^	<0.001
Condiments	11.9 (12.4)	21.6 (17.4)^A^	10.4 (10.5)^B^	8.09 (6.83)^B^	<0.001	12.8 (13.4)	21.8 (20.0)^A^	8.65 (8.54)^C^	12.0 (10.1)^B^	<0.001
Ready-to-eat meals	91.8 (94.7)	79.3 (94.5)^B^	132 (118)^A^	59.2 (37.7)^B^	<0.001	84.9 (60.4)	63.6 (58.2)^B^	97.3 (65.9)^A^	85.1 (52.0)^A^	<0.001
Fatty and salty products	9.7 (14.4)	8.93 (11.3)^B^	14.1 (19.8)^A^	5.74 (6.05)^B^	<0.001	9.47 (11.5)	8.84 (12.2)^A^	10.9 (13.4)^A^	8.44 (8.41)^A^	0.050
**Beverages**										
Milk	140 (534)	130 (378)^A^	114 (159)^A^	84.1 (128)^A^	0.130	115 (236)	101 (243)^A^	92.6 (132)^A^	117 (174)^A^	0.328
Bottled water	565 (1093)	338 (773)^B^	995 (1433)^A^	237 (545)^B^	<0.001	561 (1159)	265 (614)^B^	959 (1568)^A^	324 (721)^B^	<0.001
Tap water	1683 (1338)	1770 (1301)^A^	1593 (1293)^A^	1730 (1400)^A^	0.400	1994 (2000)	1814 (1670)^B^	1414 (1359)^B^	2693 (2455)^A^	<0.001
Fruit juice	132 (239)	72.4 (138)^B^	201 (323)^A^	95.6 (152)^B^	<0.001	133 (231)	61.1 (99.0)^B^	168 (284)^A^	138 (215)^A^	<0.001
Sweetened beverages	261 (680)	163 (515)^B^	437 (952)^A^	143 (291)^B^	<0.001	222 (464)	129 (343)^B^	307 (595)^A^	190 (348)^A,B^	0.001
Alcohol	42.5 (76.3)	64.8 (131)^A^	42.5 (59.6)^B^	30.3 (39.1)^B^	<0.001	43.8 (71.7)	60.1 (113)^A^	35.1 (50.5)^B^	43.9 (57.9)^A,B^	0.008
Hot drinks	259 (429)	559 (628)^A^	188 (352)^B^	164 (259)^B^	<0.001	286 (502)	604 (793)^A^	148 (272)^B^	250 (391)^B^	<0.001
Soy milk	23.2 (170)	25.7 (98.5)^A^	6.09 (33.8)^B^	9.58 (39.9)^B^	<0.001	16.2 (76.7)	19.3 (64.2)^A^	5.76 (36.6)^A^	15.0 (62.0)^A^	0.051

Values are means (SD, Standard Deviation). Food groups presented in this table reflect the simplified classification (see Supplementary material S1). Means with the same letter are not significantly different at alpha = 0.05 level, after Bonferroni correction for multiple comparisons.^a^Three participants were excluded from dietary groups comparison.

The *healthy diet* group had the highest daily consumption of eggs, poultry, vegetables, pulses, fruit, condiments, alcohol and hot drinks. The *Western diet* group had the highest daily consumption of cream or soy-based deserts, fats and sauces, pork, processed meat and offal, starchy vegetables, sweet products, ready-to-eat meals, fatty and salty products, bottled water, fruit juice and sweetened beverages. The *frugal diet* group had a lower daily intake of all food groups except tap water, with significant differences for bread and cereals, dairy products, red meat, seafood, vegetables, and fruit. Between-group comparisons of food group consumption adjusted for energy intake (i.e., per 2000 kcal of diet) highlighted that for the same amount of energy, the *frugal diet* group had a similar consumption pattern to the *Western diet* group for most food groups, but differed by a higher consumption of eggs and condiments and a lower consumption of pork, red meat, and processed meat. Moreover, the *frugal diet* group had similar consumption levels as the *healthy diet* group for cream and soy-based desserts, pork, red meat, and fish.

Similar differences across the three dietary groups were found in sensitivity analyzes after removing under- and over-reporters for energy intake (see Supplementary material S4).

### 3.3 Dietary indicators across dietary groups

On average, students consumed 2,098 ± 1,023 kcal/day, as shown in Table 2. The *frugal diet* group differed from the other groups with a lower macronutrient content due to lower energy intake (1,428 ± 457 kcal/day vs. 2,384 ± 1,081 kcal/day for the *healthy diet* group and 2,560 ± 958 kcal/day for the *Western diet* group).

**Table 2:** Macronutrient content, nutritional quality, environmental impact, organics and local food consumption across the three dietary groups of French university students (N = 582).

	Overall *N* = 582^a^	Healthy diet group *N* = 125	Western diet group *N* = 227	Frugal diet group *N* = 227	*p*
**Macronutrient content**					
Energy intake (kcal/day)	2099 (1023)	2384 (1081)^A^	2560 (958)^A^	1428 (457)^B^	<0.001
Protein (g/day)	88.1 (53.7)	110 (66.9)^A^	104 (44.1)^A^	56.0 (21.2)^B^	<0.001
Plant-based protein (g/day)	27.1 (13.6)	33.4 (15.8)^A^	31.5 (13.6)^A^	19.2 (6.90)^B^	<0.001
Animal-based protein (g/day)	60.0 (43.4)	75.8 (57.0)^A^	72.2 (36.6)^A^	36.4 (17.0)^B^	<0.001
Fat (g/day)	85.3 (46.0)	96.3 (51.3)^A^	106 (44.4)^A^	56.9 (20.5)^B^	<0.001
Saturated fatty acids (g/day)	36.0 (20.5)	39.6 (23.8)^B^	44.8 (19.6)^A^	24.2 (9.3)^C^	<0.001
Carbohydrate (g/day)	239 (122)	262 (117)^B^	291 (132)^A^	168 (60.7)^C^	<0.001
Fiber (g/day)	20.7 (11.5)	28.1 (15.2)^A^	23.1 (10.1)^B^	14.3 (5.7)^C^	<0.001
**Nutritional quality**					
sPNNS-GS2	−0.78 (2.84)	0.77 (2.74)^A^	−1.61 (2.80)^C^	−0.82 (2.60)^B^	<0.001
**Environmental indicators**					
GHGE (kg eCO_2_/day)	5.80 (3.86)	6.70 (4.32)^A^	7.38 (3.89)^A^	3.57 (1.75)^B^	<0.001
GHGE (kg eCO_2_/2000 kcal)	5.37 (1.67)	5.48 (1.78)^A^	5.74 (1.72)^A^	4.94 (1.46)^B^	<0.001
**Organic and local scores**					
Organic score [range (0;2)]	0.55 (0.44)	0.66 (0.46)^A^	0.55 (0.45)^A.B^	0.49 (0.42)^B^	0.002
Local score [range (0;2)]	0.55 (0.43)	0.61 (0.45)^A^	0.58 (0.42)^A.B^	0.49 (0.41)^B^	0.025
**Under and over-reporters for energy intake according to the Black method**					
Under-reporters	27% (*N* = 159)	18% (*N* = 23)	9% (*N* = 21)	50% (*N* = 114)	
Over-reporters	4% (*N* = 26)	5% (*N* = 6)	7% (*N* = 17)	0% (*N* = 0)	

Values are means (SD, Standard Deviation) Means with the same letter are not significantly different at alpha = 0.05 level, after Bonferroni correction for multiple comparisons.^a^Three participants were excluded from dietary groups comparison.

As suggested by the description of intakes by food groups, the *healthy diet* group had a higher nutritional quality (sPNNS-GS2 = 0.8 ± 2.7) than the *Western diet* group (sPNNS-GS2 = −1.6 ± 2.8) and the *frugal diet* group (sPNNS-GS2 = −0.8 ± 2.6). Sensitivity analyzes conducted using the PANDiet score, an indicator measuring the probability of adequate nutrient intake, confirmed that the *healthy diet* group had the highest level of nutritional quality (see Supplementary material S5).

The average values for GHGE were 5.8 ±3.9 kg eCO_2_/day and 5.4 ± 1.7 kg eCO_2_/2000 kcal (Table 2). The *frugal diet* group differed from the other two groups with significantly lower GHGE (3.6 ± 1.7 kg eCO_2_/day vs. 6.7 ± 4.3 for the *healthy diet* group and 7.4 ± 3.9 for the *Western diet* group), even when adjusted for 2000 kcal (4.9 ± 1.5 kg eCO_2_/2000 kcal vs. 5.5 ± 1.8 for the *healthy diet* group and 5.7 ± 1.7 kg for the *Western diet* group). The *healthy diet* group and the *Western diet* group had similar GHGE per day and per 2000 kcal. However, participants in the *healthy diet* cluster had a higher consumption of organic and locally produced food and drink products compared to the participants in the *frugal diet* cluster.

The same pattern of results across the three dietary groups was found in sensitivity analyzes after removing under- and over-reporters for energy intake (see Supplementary materials S6).

### 3.4 Participants’ characteristics across dietary groups

Comparisons of participants’ characteristics between the three dietary groups (Table 3) revealed significant differences in terms of age, number of years in university, level of physical activity, willingness to gain muscle mass, and living conditions but not in terms of sex, scholarship status and BMI.

**Table 3:** French university students’ characteristics across the three dietary groups.

	Healthy diet group *N* = 125	Western diet group *N* = 227	Frugal diet group *N* = 227	*p*
**Age**, *years*, mean (SD)	21.9 (3.14)^A^	20.9 (2.51)^B^	20.5 (2.12)^B^	<0.001
**Sex**, *female*, *n* (%)	69 (55.2)	123 (54.2)	135 (59.5)	0.498
**Scholarship status**, *with scholarship*, *n* (%)	49 (39.2)	86 (37.9)	96 (42.3)	0.622
**Nationality, French**, *n* (%)	118 (94.4)	211 (92.9)	214 (94.3)	0.801
**Number of years having been a student**, *years*, mean (SD)	3.73 (2.23)^A^	3.30 (2.23)^A.B^	3.01 (1.93)^B^	0.008
**Type of institution** *, *n* (%)				0.722
University	78 (62.4)	143 (63.0)	135 (59.5)	
Others	47 (37.6)	84 (37.0)	92 (40.5)	
**Field of studies** **, *n* (%)				0.620
Science	80 (64.0)	150 (66.1)	140 (61.7)	
Humanities	45 (36.0)	77 (33.9)	87 (38.3)	
**Highest educational qualification of parents**, *n* (%)				0.822
< High-school +2 years diploma	41 (34.2)	71 (32.6)	72 (32.7)	
High-school +2 years diploma	26 (21.7)	47 (21.6)	37 (16.8)	
High-school +3 or + 4 years diploma	20 (16.7)	36 (16.5)	45 (20.4)	
≥ High-school +5 years diploma	33 (27.5)	64 (29.4)	66 (30)	
**Place of living**, *n* (%)				0.008
Parents’ house	21 (16.8)	47 (20.9)	20 (8.8)	
Students’ house or boarding school	27 (21.6)	51 (22.7)	64 (28.3)	
Independent accommodation	77 (61.6)	127 (56.4)	142 (62.8)	
**Living alone**, *n* (%)				0.005
Yes	65 (52.0)	115 (50.7)	144 (63.4)	
No, with parents	21 (16.8)	47 (20.7)	20 (8.8)	
No, with a partner or flatmates	39 (32.1)	65 (28.6)	63 (27.8)	
**Level of physical activity**, *n* (%)				<0.001
Low	4 (3.2)	39 (17.2)	24 (10.6)	
Moderate	60 (48.0)	114 (50.2)	130 (57.3)	
High	61 (48.8)	74 (32.6)	73 (32.1)	
**Dieting status**, yes, *n* (%)	11 (8.8)	22 (9.7)	20 (8.8)	0.937
**Willingness to gain body mass**, yes, *n* (%)	30 (24.0)	28 (12.3)	22 (9.7)	<0.001
**BMI, body-mass index**, *kg/m^2^*, mean (SD)	22.5 (3.28)	22.6 (4.26)	22.3 (3.61)	0.755
**WHO classification of weight status**, *n* (%)				0.622
Underweight [BMI < 18.5 kg/m^2^]	8 (6.4)	25 (11.0)	22 (9.7)	
Normal weight [18.5 ≤ BMI ≤ 24.9]	93 (74.4)	153 (67.4)	157 (69.2)	
Overweight or obese [BMI ≥ 25]	24 (19.2)	49 (21.6)	48 (21.1)	
**Declared diet**, *n* (%)				0.066
Omnivore	76 (60.8)	165 (72.7)	151 (66.5)	
Flexitarian, pesco-vegetarian, ovo-lacto-vegetarian or vegan diet	49 (39.2)	62 (27.3)	76 (33.5)	

Data for the whole group are reported in Supplemental material S5. *Type of institution responses grouped together in “others”: “engineering school,” “business school,” “art school,” “higher school preparatory classes,” “technician school” and “others”. **“Field of studies” responses grouped together in “Science”: “Industry,” “Health” and “Sciences” and “Humanities”: “art,” “business,” “law,” “teaching,” “humanities and languages,” “social sciences” and “political science”.

Participants in the *healthy diet* group were on average older students (21.9 ± 3.1 years) who had been studying for a longer period of time (3.7 ± 2.2 years). They were also more likely to be living with a partner or flatmates (32%), to have a high level of physical activity (49%) or to be willing to gain muscle mass (24%). The *Western diet* group differed from the *frugal diet* group by having a higher proportion of students with a low level of physical activity and who lived with their parents. Students in the *frugal diet* group were more likely to live alone.

The same pattern of results across the three dietary groups was found in sensitivity analyzes after removing under- and over-reporters. Age, place of residence, level of physical activity, and willingness to gain body mass variables remained statistically significant (see Supplementary materials S7). For the number of years having been a student and living alone variables, differences were no longer significant but the trend remained the same.

## 4 Discussion

The present study highlighted the diversity of food consumption patterns that coexist in the French student population. Three dietary groups that varied in nutritional quality and environmental impact were identified through clustering analysis. The *Western diet* group (40% of the sample) had the poorest nutritional quality and the highest environmental impact. The *healthy diet* group (20%) had a similarly high environmental impact but had the highest nutritional quality. The *frugal diet* group (40%), with the lowest energy intake, had an intermediate nutritional quality and the lowest environmental impact. Students in these groups differed in age, level of physical activity, willingness to gain muscle mass, housing, and living conditions.

The identification of three dietary groups brought nuances to the general description of students’ diets as unhealthy and unsustainable (7). Although in line with previous literature (6–9), we found that as a whole, French students have diets with relatively low nutritional quality, with a sPNNS-GS2 score of −0.8 ± 2.8 compared to an average score of 1.2 ± 2.6 in a convenience sample of adults recruited in the same geographic area as students from the present study (28) and scores of −0.3 ± 3.6 and 2.1 ± 3.1 in samples of French men and women, respectively (38). Regarding environmental impact, students’ diets had an equivalent carbon footprint (5.4 ± 1.7 kg eCO2/2000 kcal) as what had been found for adults in a previous study using the exact same calculation method (same FFQ and same GHGE data) (5.6 ± 1.5 kg eCO2/2000 kcal) (28).

Other studies have identified similar dietary patterns as in the present study (24, 44), suggesting that the dietary groups that emerged in this study may not be specific to the French student population. Two of the four dietary profiles identified in a United Kingdom-based study with a convenience sample of 1,448 university students can be compared with the groups described in this study (24). The ‘health-conscious’ profile from the United Kingdom study, characterized by food groups such as fish, nuts, eggs, fruits, and vegetables, could be linked to the *healthy diet* group. The ‘convenience, red meat & alcohol’ profile resonates with our *Western diet* group. Alcohol consumption follows a different pattern in our sample from that of the United Kingdom study, with the *healthy diet* group showing higher intakes. Despite this difference, and because the sPNNS-GS2 score takes into account alcohol consumption (38), this group is still characterized by a higher overall nutritional quality compared to other dietary groups. However, a ‘vegetarian’ profile, characterized by the consumption of pulses, nuts and vegetables and an avoidance of meat and fish, emerged in the United Kingdom study but not in the present study (24), possibly because students declaring that they excluded meat and fish from their diet represented only 7.9% of our sample. This ‘vegetarian’ profile may be embedded in the *healthy diet* group, which seemed to have higher percentages of flexitarians, vegetarians and vegans. A fourth profile was identified in the United Kingdom-based study, without the equivalent found in our study: the ‘snacking’ profile, which was associated with the consumption of savory and fatty foods, sweetened beverages, and sweet products, which may be due to cultural differences regarding snacking between France and the United Kingdom (45). Finally, the *frugal diet* group was not identified in the sample of United Kingdom university students. The lower energy intake in the *frugal diet* group may raise questions about whether it may reflect the reliability of the results from the food frequency questionnaire or actual disparities in energy intake. Even after excluding 158 under-reporters for energy intake using the cut-off points proposed by Black, this group still showed significantly lower average energy intake (1740 ± 392 kcal/day) compared to the *healthy diet* group (2,506 ± 836 kcal/day) and the *Western diet* group (2,511 ± 634 kcal/day). This suggests that the observed difference may actually reflect distinct eating behaviors. The observation of this cluster with particularly low energy intake could be linked to the overall difficult economic situation at the time of the study as an increased prevalence of food insecurity in the student population was reported at this time following the COVID-19 pandemic (46).

Even if theoretical diets optimizing both nutritional quality and environmental impact have been described in the literature (e.g., EAT Lancet commission diet (19)), individuals adopting a healthy and low-carbon diet were not widespread in our sample of French students. On the one hand, the dietary group with the lowest environmental impact (the *frugal diet* group) had diets with intermediate nutritional quality. In contrast, the dietary group with the best nutritional quality (*healthy diet* group) had the highest environmental impact. The food groups that characterized each dietary group explained these results. We noticed that some food groups widely consumed by students have asymmetric effects on nutritional quality and environmental impact. For example, poultry and fish have favorable nutritional profiles (38) but are animal-based products that have a relatively high environmental impact (47). In contrast, some plant-based foods with a relatively low environmental impact are not healthy (i.e., sugary, salty or fatty foods such as cakes, sweetened beverages, or fried potatoes) (48). In the *frugal diet* group, the relatively low nutritional quality and low environmental impact can be explained by the low consumption of both meat and healthy plant-based foods (i.e., pulses, vegetables, and fruit). In the *healthy diet* group, we observed a high consumption of animal-based foods with a high environmental impact but good nutritional quality, such as poultry, fish, eggs, and dairy products. The consumption of these food groups resonates with the fact that this dietary group is composed of students who more often declared wanting to gain muscle mass and with a higher level of physical activity. In our sample, a higher level of physical activity was associated with higher consumption of meat (data not shown). Students in the *healthy diet* group might have been more focused on their health than on the environmental impact of their diets, resulting in the choice of familiar and healthy animal-sourced foods rather than plant-based alternatives. Students in this cluster also declare a high consumption of organic food products. Even if organic farming has positive impacts on the environment (49), especially the biodiversity (50), health is the first motive that explains organic food consumption (51).

The three identified dietary groups differed according to students’ age, level of physical activity, willingness to gain muscle mass, as well as housing and living conditions. Contrary to previous studies reporting that more women have a healthy diet (9, 24, 52), sex did not discriminate the dietary groups in the present study. The results regarding physical activity levels were similar to those described in the literature, with students exercising more often also having healthier diets (53). Students in the *healthy diet* group were older than those in the other two groups, which resonates with the positive association between age and nutritional quality reported in previous studies (6, 7, 24, 54). This association may be explained by the fact that when students enter university, they must choose when, what, and with whom to eat for the first time. They are gaining independence in their food choices while lacking knowledge and skills for food shopping, storing and cooking (7, 24, 55). Longitudinal studies are needed to better understand whether the association between age and nutritional quality can be explained by individual factors such as knowledge, food literacy, and cooking skills, and/or structural factors, such as financial resources or lack of cooking equipment. Previous studies have highlighted that leaving the family house decreases the nutritional quality of students’ diets (6, 7, 56, 57). However, in our study, students with the lowest nutritional quality (i.e., in the *Western diet* group) were most likely to live with their parents. We hypothesized that students living at the family house may have more financial resources to eat more frequently outside of home than students living alone, who are also more likely to adopt a *frugal diet*. Indeed, the food offered on campus is known to negatively influence the nutritional quality of students’ diets (26, 58). Finally, as one can wonder whether the *frugal diet* is chosen or adopted under the strain of financial constraints, further studies are needed to describe students’ diets while considering the socioeconomic dimension of diets (food cost and food expenditure).

This study provides a better understanding of the specific margins of progress in terms of improving nutritional quality and limiting the environmental impact among students by differentiating several dietary groups. The sociodemographic characteristics related to each dietary group allows to identify the subgroups of students to target in priority in public health interventions. Younger students and those living alone are the most represented in the frugal diet cluster and would particularly benefit from interventions promoting healthy and sustainable diets, especially from an improvement of nutritional quality. Interventions targeting the healthy diet cluster should focus on the reduction of meat consumption to limit the environmental impact of their diets. This includes promoting plant-based alternatives like pulses as part of healthy diets. Interventions could be carried out by local communities that participate in shaping students’ food environments such as university catering (59). According to the present study, a large number of students rely on the university food system as they regularly eat at university catering facilities (54%) and/or live in university accommodations (26%). This is all the more important as the university period can be considered as an opportunity to implement public health interventions before the leap to independent life where contexts are more varied and policy implementation more difficult.

### 4.1 Strengths and limitations

This is the first study to provide a detailed description of food consumption patterns in a sample of French students. A quota sampling method was used to recruit a representative sample of French students for age, sex and scholarship status which is a major strength of this study. It is though important to acknowledge that the dataset was only obtained from participants studying in the city of Dijon, thereby potentially impeding the generalizability of the study results to all of French students. Another limitation of this study is relevant to data collection methodology. Indeed, the FFQ is sensitive to reporting bias and memory bias which may affect the robustness of the results. However, the questionnaire was previously validated (34), and sensitivity analyzes excluding under- and over-reporters for energy intake led to consistent results.

## 5 Conclusion

Overall, French students have diets with lower nutritional quality and similar environmental impacts compared to French adults. However, while some students reported high-energy diets with poor nutritional quality (40%) classified as *Western diet*, we also identified a dietary group with significantly lower energy (40%) classified as *frugal diet* and a group with significantly higher nutritional quality (20%) classified as *healthy diet*. Students adopting these different food consumption patterns were notably discriminated by age and living conditions, highlighting potential skill-related and structural influences on students’ dietary choices. In particular, younger students were more likely to have diets with worse nutritional quality, and those living alone were more likely to have diets with lower energy content. In this study, none of the dietary groups optimized both nutritional quality and environmental impact simultaneously, a key factor in promoting sustainable eating habits. Indeed, the *healthy diet* group presents high nutritional quality but also high environmental impact. These findings are crucial for future public health efforts which could be undertaken by university catering facilities as a substantial number of students regularly use them. First, the results from this study emphasize the importance of harmonizing nutrition and environmental concerns. This includes promoting plant-based alternatives like pulses over animal-based products like poultry or fish in healthier diets, which is not currently the case in French food-based dietary guidelines. Second, the results from this study give practical information regarding the students to target in future public health interventions. Younger students with lower diet quality could benefit the most from interventions promoting healthy and sustainable diets.

## Data availability statement

The datasets presented in this study can be found in online repositories. The names of the repository/repositories and accession number(s) can be found: https://osf.io/z53na/.

## Ethics statement

The studies involving humans were approved by Ethical evaluation committee for research of Inserm. The studies were conducted in accordance with the local legislation and institutional requirements. The participants provided their written informed consent to participate in this study.

## Author contributions

LA: Conceptualization, Data curation, Formal analysis, Investigation, Methodology, Software, Writing – original draft, Writing – review & editing. SN: Conceptualization, Formal analysis, Funding acquisition, Methodology, Project administration, Resources, Supervision, Validation, Visualization, Writing – review & editing. BL-G: Conceptualization, Formal analysis, Methodology, Resources, Software, Validation, Writing – review & editing. LM: Conceptualization, Formal analysis, Methodology, Project administration, Resources, Software, Supervision, Validation, Visualization, Writing – review & editing.

## Footnotes

^1^ http://www.qualtrics.com

^2^https://osf.io/z53na/
