# Supplementary material for: Identification of three dietary groups in French university students and their associations with nutritional quality and environmental impact
Source: Front Nutr. 2023 Dec 20;10:1323648. doi: 10.3389/fnut.2023.1323648 (PMC10771388; doi:10.3389/fnut.2023.1323648)
Supplement: Supplementary file 1 [file Data_Sheet_1.docx]

Supplementary Material

**S1. Food and beverage groups derived from the 125 FFQ items**

| **^[[1]](#footnote-1)^Items extracted from the FFQ** | **Food and beverage classification (N=39 groups)** | **Simplified food and beverage classification (N=26 groups)** |
| --- | --- | --- |
| ***FOOD*** | | |
| Bread (white) | Bread and dry bread | **Bread and cereals** |
| Crispbread, crackers, toasted bread |  |  |
| Whole and seed breads | Whole bread and dry bread |  |
| Pasta (macaroni, spaghetti...) | Pasta, rice, wheat and other cereals |  |
| White rice |  |  |
| Semolina, wheat (tabbouleh, as a side dish other than in couscous, Ebly) |  |  |
| Other starchy foods (quinoa, manioc, plantain, yam,...) |  |  |
| Cottage cheese or 0% fat yoghurt (plain, with fruit, etc.) | Yoghurts | **Dairy products** |
| Cottage cheese or yoghurt with 20%, 30% or 40% fat |  |  |
| Emmental, Gruyère, Comté, Beaufort - in pieces | Cheese |  |
| Emmental, Gruyère, Comté, Beaufort - grated on dishes (pasta, rice...) |  |  |
| Roquefort cheese, blue cheese |  |  |
| Other types of cheese (camembert, brie...) |  |  |
| Desserts (cream desserts such as Danette, liégeois, mousses, flans, etc.) | Entremets and cream desserts | **Cream or soy-based desserts** |
| Ice cream | Ice creams |  |
| Sorbets |  |  |
| Soya desserts or soya yoghurt | Soy substitutes |  |
| Butter (added to bread, toast, on pasta...) | Animal fats | **Fats and sauces** |
| Sour cream |  |  |
| Margarine | Vegetable fats |  |
| Mayonnaise | Sauces |  |
| Vinaigrette sauce (with raw vegetables...) |  |  |
| Ketchup |  |  |
| Fried eggs, omelette | Eggs | **Eggs** |
| Poached, hard-boiled or boiled eggs |  |  |
| Beef (except minced beef) | Red meat | **Red meat** |
| Minced beef |  |  |
| Veal |  |  |
| Lamb |  |  |
| Poultry (chicken, turkey...), rabbit | Poultry | **Poultry** |
| Pork meat (except cold cuts) | Pork | **Pork** |
| Dry sausage (or salami) | Processed meat | **Processed meat and offal** |
| Cervelas or mortadella |  |  |
| Pâté or rillettes |  |  |
| Ham (white, raw, bacon...) |  |  |
| Fresh or smoked sausages (including Merguez) |  |  |
| Liver (heifer, poultry, others) | Offal |  |
| Beef tongue, innards, blood sausage, andouillettes, sweetbreads, kidneys |  |  |
| Fish (cod, pollack, whiting, sole, trout...) fresh or frozen (except breaded fish) | Fish & seafood | **Fish &seafood** |
| Fish in oil (tuna, sardines...) |  |  |
| Smoked fish (salmon, trout) |  |  |
| Salted or pickled fish (cod, herring, anchovies) |  |  |
| Breaded fish |  |  |
| Shellfish (mussels, oysters, scallops) |  |  |
| Shellfish (shrimp, crab) |  |  |
| Vegetable soup | Vegetables | **Vegetables** |
| Green beans |  |  |
| Endives, spinach, watercress |  |  |
| Leeks |  |  |
| Cabbage (green, cauliflower, Brussels sprouts, broccoli...) |  |  |
| Cooked carrots |  |  |
| Zucchinis, eggplants, peppers, cooked tomatoes (ratatouille...) |  |  |
| Peas |  |  |
| Artichokes, fennel, asparagus, celery |  |  |
| Mushrooms |  |  |
| Maize |  |  |
| Pumpkin, sweet potatoes |  |  |
| Green salad, lettuce, rocket, spinach, watercress |  |  |
| Grated carrots |  |  |
| Avocado |  |  |
| Other raw vegetables (tomatoes, beetroot, cabbage, cucumber, radish...) |  |  |
| Pulses (lentils, beans, chickpeas, broad beans, etc.) | Pulses | **Pulses** |
| Boiled or jacket potatoes | Potatoes and other tubers | **Starchy vegetables** |
| Mashed potatoes |  |  |
| Gratin dauphinois |  |  |
| Hash browns or fried potatoes | Fried potatoes |  |
| Chips |  |  |
| Apricots, peaches, plums, cherries | Fresh and dried fruits | **Fruit** |
| Strawberries, raspberries |  |  |
| Grapes |  |  |
| Melon, watermelon |  |  |
| Bananas |  |  |
| Kiwis |  |  |
| Citrus fruits (oranges, mandarins, grapefruits, etc.) |  |  |
| Apples, pears |  |  |
| Exotic fruits (pineapples, mangoes, litchis, guavas...) |  |  |
| Dried fruit (apricots, dates, figs, prunes, etc.) |  |  |
| Nuts (walnuts, hazelnuts, almonds, etc.) | Nuts | **Nuts** |
| Pastries (croissants, pains au chocolat, etc.) | Cakes | **Sweet products** |
| Pie (fruit, custard...) |  |  |
| Brioche, cake, pound cake |  |  |
| Biscuits (pure butter, dry, with jam, filled, chocolate...) |  |  |
| Pastry cakes (chocolate, cream, etc.) |  |  |
| Chocolate bars (Mars, Bounty...) |  |  |
| Cereal bars (Granny...) |  |  |
| Breakfast cereals (corn flakes, chocolate cheerio’s, puffed cereals, muesli...) | Breakfast cereals and cereal bars |  |
| Chocolate (dark, milk, hazelnut, etc.) | Sugary foods |  |
| Chocolate spread (Nutella...) |  |  |
| Honey, jam or marmalade |  |  |
| Cocoa or chocolate powder |  |  |
| Sweets |  |  |
| Ravioli, lasagne, filled pasta | Mixed dishes | **Ready-to-eat meals** |
| Cassoulet |  |  |
| Couscous |  |  |
| Paella |  |  |
| Sauerkraut (with cold cuts) |  |  |
| Chili con carne |  |  |
| Ready-made fish dishes |  |  |
| Low-fat ready meals |  |  |
| Puff pastry (croque-monsieur, filled pancakes, ham croissants...) | Sandwiches, pizzas, pies |  |
| Savoury tarts (quiche...) |  |  |
| Sandwiches |  |  |
| Pizza |  |  |
| Burgers, kebabs, tacos |  |  |
| Salted peanuts | Fatty and salty products | **Fatty and salty products** |
| Savoury appetizer cakes |  |  |
| Salty snack cakes chips |  |  |
| Garlic | Condiments, herbs and spices | **Condiments** |
| Onion, shallots, chives (or spring onion or civet) |  |  |
| Aniseed, dill |  |  |
| Spices such as cinnamon, cloves, cumin, nutmeg, saffron, caraway, curry |  |  |
| Gherkins, capers, pickled onions |  |  |
| Ginger |  |  |
| Fresh herbs such as basil, chervil, coriander, parsley, tarragon |  |  |
| Dry herbs such as bay leaves, thyme, rosemary, savoury, oregano |  |  |
| Mint |  |  |
| Mustard |  |  |
| Pepper |  |  |
| Chilli, Tabasco, chilli sauce, Espelette pepper, paprika... |  |  |
| Vanilla (in ice creams, desserts, yoghurts, cream desserts, etc.) |  |  |
| Soy sauce |  |  |
| ***BEVERAGES*** | | |
| Water in bottles | Bottled water | **Bottled water** |
| Tap water | Tap water | **Tap water** |
| Whole milk | Milk | **Milk** |
| Semi-skimmed milk |  |  |
| Skimmed milk |  |  |
| Soya beverages | Soy milk | **Soy milk** |
| Orange juice, grapefruit juice, pineapple juice, apple juice, grape juice... | Fruit juice | **Fruit juice** |
| Syrup or flavoured water | Sugary drinks | **Sugary drinks** |
| Cola (such as Coca-Cola or Pepsi), lemonade or soda (such as Sprite, Fanta, etc.), non-light energy drinks (Red bull, Crazy tiger, etc.) |  |  |
| "Light" cola (such as Coca-Cola or Pepsi), light lemonade or light soda (such as Sprite, Fanta, etc.), light energy drinks (Red bull, Crazy tiger, etc.) |  |  |
| Cider or beer | Beer and wine | **Alcohol** |
| Wine |  |  |
| Aperitifs (pastis, cherry, port, martini...) | Spirits |  |
| Spirits (whisky, gin, vodka, premix…) |  |  |
| Coffee (including decaffeinated) | Hot drinks | **Hot drinks** |
| Tea |  |  |

**S2. Survey flow**

**
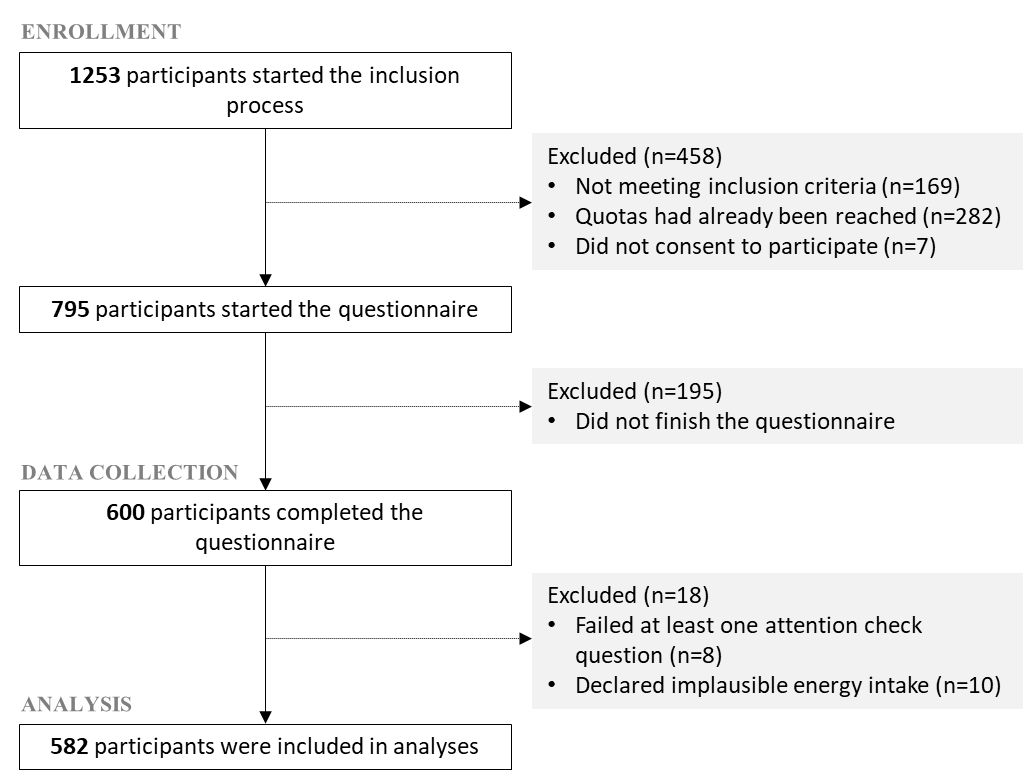
**

**S3. French university student’ characteristics (N=582)**

| **Age**, *years*, mean (SD) | 21.0 (2.6) |
| --- | --- |
| **Gender**, *female*, n (%) | 329 (56%) |
| **Scholarship status**, *with scholarship*, n (%) | 233 (40%) |
| **Nationality**, *French*, n (%) | 546 (94%) |
| **Number of years having been a student**, *years*, mean (SD) | 3.3 (2.1) |
| **Type of institution** ^a^**,** n (%) |  |
| University | 359 (61.7%) |
| Others | 223 (38.3%) |
| **Field of studies** ^b^, n (%) |  |
| Science | 372 (63.9%) |
| Humanities | 210 (36.1%) |
| **Highest educational qualification of parents**, n (%) |  |
| < High-school +2 years diploma | 184 (31.6%) |
| High-school +2 years diploma | 110 (18.9%) |
| High-school +3 or +4 years diploma | 101 (17.3%) |
| ≥ High-school +5 years diploma | 165 (28.4%) |
| Do not know | 22 (3.8%) |
| **Place of living**, n (%) |  |
| Parents’ house | 88 (15.1%) |
| Students’ house | 133 (23.9%) |
| Boarding school | 10 (1.7%) |
| Independent accommodation | 348 (59.8%) |
| Others | 3 (0.5%) |
| **Living alone**, n (%) |  |
| Yes | 327 (56.2%) |
| No, with parents | 88 (15.1%) |
| No, with a partner or flatmates | 167 (28.7%) |
| **Level of physical activity**, n (%) |  |
| Low | 67 (11.5%) |
| Moderate | 306 (52.6%) |
| High | 209 (35.9%) |
| **Dieting status**, yes, n (%) | 53 (9.1%) |
| **Willingness to gain muscle mass**, yes, n (%) | 80 (13.7%) |
| **BMI**, **body-mass index**, *kg/m^2^*, mean (SD) | 22.4 (3.8) |
| **WHO classification of weight status**, n (%) |  |
| Underweight [BMI < 18.5 kg/m²] | 55 (9.3%) |
| Normal weight [18.5 ≤ BMI ≤ 24.9] | 405 (69.6%) |
| Overweight [25 ≤ BMI ≤ 29.9] | 97 (16.7%) |
| Obese [BMI ≥ 30] | 25 (4.3%) |
| **Declared diet**, n (%) |  |
| Omnivore | 393 (67.5%) |
| Flexitarian | 143 (24.6%) |
| Pesco-vegetarian | 19 (3.3%) |
| Ovo-lacto-vegetarian | 25 (4.3%) |
| Vegan | 2 (0.3%) |

^a^ Type of institution responses grouped together in “**others**”: “engineering school”, “business school”, “art school”, “higher school preparatory classes”, “technician school” and “others”

^b^ “Field of studies” responses grouped together in “**Science**”: “Industry”, “Health” and “Sciences” and “**Humanities**”: “art”, “business”, “law”, “teaching”, “humanities and languages”, “social sciences” and “political science”)

**S4.** **Food and beverage consumptions in grams per day and for an isocaloric diet (/2000 kcal) across the three dietary groups of French university students after removing under and over reporters for energy intake (sensitivity analyses)**

|  | **Food groups in g/day and beverages in ml/day** | | | | | **Food groups in g/2000 kcal and beverages in ml/2000 kcal** | | | | |
| --- | --- | --- | --- | --- | --- | --- | --- | --- | --- | --- |
|  | **Overall** | **Healthy diet group** | **Western diet group** | **Frugal diet group** | **p** | **Overall** | **Healthy diet group** | **Western diet group** | **Frugal diet group** | **p** |
|  | N=398 | N=96 | N=189 | N=113 |  | N=398 | N=96 | N=189 | N=113 |  |
| **Food groups** |  |  |  |  |  |  |  |  |  |  |
| Bread and cereal | 292 (219) | 355 (279) ^A^ | 296 (203) ^A,B^ | 233 (166) ^B^ | <0.001 | 248 (146) | 266 (150) ^A^ | 230 (124) ^A^ | 262 (170) ^A^ | 0.068 |
| Dairy products | 98.6 (87.3) | 114 (94.6) ^A^ | 102 (86.0) ^A,B^ | 78.7 (79.8) ^B^ | 0.009 | 85.9 (72.6) | 90.7 (65.0) ^A^ | 81.0 (64.4) ^A^ | 89.9 (89.7) ^A^ | 0.442 |
| Dairy and soy-based desserts | 36.1 (45.3) | 27.3 (40.3) ^B^ | 45.8 (50.8) ^A^ | 27.3 (35.4) ^B^ | <0.001 | 33.0 (43.0) | 23.0 (33.1) ^B^ | 39.3 (50.0) ^A^ | 31.1 (35.8) ^A,B^ | 0.008 |
| Fats and sauces | 20.5 (15.3) | 17.4 (14.4) ^B^ | 24.0 (16.2) ^A^ | 17.5 (13.3) ^B^ | <0.001 | 19.0 (14.7) | 15.2 (13.1) ^B^ | 20.4 (15.5) ^A^ | 20.0 (14.1) ^A^ | 0.013 |
| Egg | 51.9 (77.1) | 83.5 (114) ^A^ | 41.7 (58.1) ^B^ | 42.3 (57.3) ^B^ | <0.001 | 42.9 (53.6) | 60.1 (63.8) ^A^ | 31.6 (39.3) ^B^ | 47.2 (60.5) ^A,B^ | <.0001 |
| Red meat | 39.8 (49.2) | 39.2 (47.7) ^A^ | 50.0 (54.8) ^A^ | 23.4 (34.2) ^B^ | <0.001 | 32.0 (34.7) | 29.0 (34.3) ^A,B^ | 37.4 (35.5) ^A^ | 25.5 (32.5) ^B^ | 0.009 |
| Poultry | 34.0 (61.3) | 68.7 (111) ^A^ | 27.1 (26.3) ^B^ | 16.1 (18.1) ^B^ | <0.001 | 26.5 (36.6) | 46.0 (61.6) ^A^ | 21.8 (20.6) ^B^ | 17.7 (19.4) ^B^ | <.0001 |
| Pork | 12.7 (19.5) | 8.71 (14.1) ^B^ | 18.5 (23.9) ^A^ | 6.53 (10.6) ^B^ | <0.001 | 10.5 (15.3) | 6.82 (10.8) ^B^ | 14.1 (17.8) ^A^ | 7.48 (12.4) ^B^ | <.0001 |
| Processed meat and offal | 22.8 (25.6) | 17.2 (17.5) ^B^ | 31.3 (31.2) ^A^ | 13.3 (13.7) ^B^ | <0.001 | 19.5 (20.2) | 14.2 (14.2) ^B^ | 24.6 (23.5) ^A^ | 15.6 (16.2) ^B^ | <.0001 |
| Fish and seafood | 24.7 (34.1) | 31.1 (51.0) ^A^ | 28.6 (29.7) ^A^ | 12.7 (14.9) ^B^ | <0.001 | 20.6 (24.7) | 23.4 (33.3) ^A^ | 22.8 (22.9) ^A^ | 14.7 (17.1) ^B^ | 0.010 |
| Vegetables | 203 (215) | 331 (331) ^A^ | 184 (157) ^B^ | 124 (92.8) ^C^ | <0.001 | 185 (185) | 287 (271) ^A^ | 155 (143) ^B^ | 147 (113) ^B^ | <.0001 |
| Pulses | 24.5 (55.7) | 50.2 (102) ^A^ | 16.9 (24.4) ^B^ | 15.2 (20.7) ^B^ | <0.001 | 22.5 (48.1) | 43.9 (85.9) ^A^ | 13.6 (19.6) ^B^ | 19.3 (27.2) ^B^ | <.0001 |
| Starchy vegetables | 54.3 (49.3) | 45.8 (34.7) ^B^ | 69.0 (60.0) ^A^ | 37.0 (28.6) ^B^ | <0.001 | 84.4 (60.5) | 38.5 (30.3) ^B^ | 56.5 (49.8) ^A^ | 41.1 (28.0) ^B^ | <.0001 |
| Fruits | 146 (150) | 226 (185) ^A^ | 128 (131) ^B^ | 109 (120) ^B^ | <0.001 | 131 (138) | 185 (154) ^A^ | 106 (125) ^B^ | 126 (132) ^B^ | <.0001 |
| Sweet products | 95.5 (55.3) | 78.6 (45.2) ^B^ | 107 (60.7) ^A^ | 89.6 (49.0) ^B^ | <0.001 | 86.7 (46.7) | 66.2 (38.2) ^C^ | 86.7 (41.2) ^A^ | 104 (54.5) ^A^ | <.0001 |
| Condiments | 12.4 (12.5) | 21.9 (17.1) ^A^ | 9.58 (9.19) ^B^ | 8.99 (7.70) ^B^ | <0.001 | 11.5 (11.9) | 19.4 (17.0) ^A^ | 7.81 (7.22) ^B^ | 10.8 (9.60) ^A^ | <.0001 |
| Ready-to-eat meals | 99.5 (91.6) | 75.9 (74.8) ^B^ | 130 (110) ^A^ | 68.0 (40.9) ^B^ | <0.001 | 84.4 (60.5) | 61.0 (55.3) ^B^ | 99.5 (67.1) ^A^ | 78.9 (44.1) ^B^ | <.0001 |
| Fatty and salty products | 10.5 (13.5) | 9.41 (12.2) ^A,B^ | 13.5 (16.2) ^A^ | 6.51 (7.15) ^B^ | <0.001 | 9.54 (12.3) | 8.62 (12.8) ^A^ | 11.0 (13.7) ^A^ | 7.81 (8.59) ^A^ | 0.060 |
| **Beverages** |  |  |  |  |  |  |  |  |  |  |
| Milk | 124 (249) | 159 (426) | 118 (158) | 106 (151) | 0,274 | 108 (182) | 119 (269) ^A^ | 97.7 (138) ^A^ | 115 (156) ^A^ | 0.560 |
| Bottled water | 636 (1169) | 391 (840) ^B^ | 971 (1440) ^A^ | 284 (628) ^B^ | <0.001 | 579 (1140) | 282 (601) ^B^ | 875 (1428) ^A^ | 335 (770) ^B^ | <.0001 |
| Tap water | 1633 (1329) | 1670 (1215) | 1608 (1308) | 1645 (1462) | 0.928 | 1554 (1418) | 1486 (1213) ^B^ | 1361 (1246) ^B^ | 1936 (1749) ^A^ | 0.002 |
| Fruit juice | 149 (262) | 66.4 (95.8) ^B^ | 209 (338) ^A^ | 118 (176) ^B^ | <0.001 | 139 (247) | 57.9 (90.6) ^B^ | 176 (301) ^A^ | 145 (222) ^A^ | <.0001 |
| Sweetened beverages | 261 (599) | 149 (375) ^B^ | 355 (765) ^A^ | 199 (176) ^A,B^ | 0.010 | 218 (458) | 126 (323) ^A^ | 260 (525) ^A^ | 226 (428) ^A^ | 0.063 |
| Alcohol | 47.4 (86.9) | 75.8 (146) ^A^ | 43.8 (61.6) ^B^ | 29.3 (35.9) ^B^ | <0.001 | 41.3 (72.0) | 64.2 (120) ^A^ | 34.1 (47.5) ^B^ | 34.0 (43.0) ^B^ | 0.001 |
| Hot drinks | 267 (434) | 576 (590) ^A^ | 179 (336) ^B^ | 151 (274) ^B^ | <0.001 | 251 (426) | 551 (615) ^A^ | 140 (263) ^B^ | 182 (325) ^B^ | <.0001 |
| Soy milk | 12.7 (63.9) | 29.9 (111) ^A^ | 5.80 (32.3) ^B^ | 9.62 (43.4) ^B^ | 0.009 | 11.5 (53.5) | 21.4 (71.7) ^A^ | 5.95 (38.7) ^A^ | 12.3 (56.1) ^A^ | 0.070 |

Food groups presented in this table reflect the simplified classification with some food groups merged together (see **supplementary materials S1**).

Values are means (SD). Means with the same letter are not significantly different at alpha=0.05 level, after Bonferroni correction for multiple comparisons.

**S5. Nutritional quality across dietary groups measured with the PANDiet scoring system among a sample of French University students (n=582)**

|  | **Overall** | **Healthy diet group** | **Western diet group** | **Frugal diet group** | **p** |
| --- | --- | --- | --- | --- | --- |
|  | N=582^a^ | N=125 | N=227 | N=227 |  |
| PANDiet ^b^ | **57.5** (8.6) | **60.3** (9.3) ^A^ | **56.4** (8.5)^B^ | **57.3** (8.1)^B^ | <0.001 |
| AS | **57.6** (20.1) | **66.7** (16.8) ^A^ | **66.8** (15.6) ^A^ | **43.3** (17.4)^B^ | <0.001 |
| MS | **57.4** (22.7) | **53.8** (23.0)^B^ | **46.0** (20.0)^C^ | **71.2** (16.9)^A^ | <0.001 |

Values are means (SD). Means with the same letter are not significantly different at alpha=0.05 level, after Bonferroni correction for multiple comparisons.

^a^ Three participants were excluded from dietary groups comparison

^b^ The PANDiet scoring system computes the average of two sub-scores: the adequacy sub-score (AS) and the moderation sub-score (MS). The AS sub-score is the mean of the probabilities of adequacy for 23 protective nutrients (protein, total fat, LA, ALA, DHA, EPA+DHA, fibre, vitamin A, thiamin, riboflavin, niacin, panthatenic acid, vitamin B6, folate, vitamin b12, vitamin C, vitamin D, vitamin E, calcium, iodine, magnesium, phosphorus, potassium and bioavailable iron). Data for copper, manganese, selenium and zinc were not available in the nutritional database we used. MS is the average of the probabilities of adequacy for six nutrients which consumption should be limited (protein, total fat, SFA, cholesterol, sugars and sodium). Each sub-score was multiplied by 100 and therefore, the PANDiet score ranged from 0 to 100. The higher the score and the sub-scores, the higher the nutrient adequacy. Reference values and variability for the probability calculation used were those listed by de Gavelle et al 2018.

**S6. Macronutrient content, nutritional quality, environmental impact, organic and local food consumption across dietary groups after removing under and over reporters for energy intake (sensitivity analyses) among a sample of French university students**

| **Mean (SD)** | **Total**  N=398 | **Healthy diet group** | **Western diet group** | **Frugal diet group** | **p** |
| --- | --- | --- | --- | --- | --- |
|  |  | N=96 | N=189 | N=113 |  |
| **Macronutrient content** |  |  |  |  |  |
| Energy intake (kcal/day) | 2291 (743) | 2506.4 (836.3) ^A^ | 2511.0 (683.9) ^A^ | 1740.4 (391.7) ^B^ | <0,001 |
| Protein (g/day) | 95.9 (44.3) | 115.4 (57.1) ^A^ | 103.4 (38.4) ^A^ | 66.8 (21.4) ^B^ | <0,001 |
| Plant-based protein (g/day) | 29.8 (11.4) | 35.0 (13.7) ^A^ | 31,1 (10.7) ^B^ | 23.3 (6.3) ^C^ | <0,001 |
| Animal-based protein (g/day) | 65.4 (37.8) | 78.9 (49.8) ^A^ | 71.9 (33.6) ^A^ | 43.0 (18.4) ^B^ | <0,001 |
| Fat (g/day) | 93.1 (35.7) | 100.2 (37.3) ^A^ | 104,0 (35.3) ^A^ | 68.7 (20.1) ^B^ | <0,001 |
| Saturated fatty acids (g/day) | 39.3 (15.9) | 41.0 (16.0) ^A^ | 44,3 (16.3) ^A^ | 29.6 (9.5) ^B^ | <0,001 |
| Carbohydrate (g/day) | 261 (90.9) | 278.3 (92.0) ^A^ | 283.1 (96.6) ^A^ | 209,4 (52.1) ^B^ | <0,001 |
| Fibre (g/day) | 22.8 (10.3) | 29.1 (13.7) ^A^ | 22.9 (8.5) ^B^ | 17.3 (5.4) ^C^ | <0,001 |
| **Nutritional quality** |  |  |  |  |  |
| sPNNS-GS2 adjusted for energy | -1.1 (3.0) | 0.6 (2,9) ^A^ | -1,9 (2,9) ^B^ | -1.2 (2,7) ^B^ | <0,001 |
| PANDiet | 58.2 (8.70) | 60.5 (8.7) ^A^ | 56.7 (8.5) ^B^ | 58.8 (8,5) ^A,B^ | 0,001 |
| AS | 64.1 (16.7) | 70.9 (13.7) ^A^ | 67,9 (14,5) ^A^ | 51.9 (16.2) ^B^ | <0,001 |
| MS | 52.4 (20.4) | 50.2 (20.5) ^B^ | 45.6 (17.8) ^B^ | 65.8 (18.2) ^A^ | <0,001 |
| **Environmental indicators** |  |  |  |  |  |
| GHGE per day | 6.31 (3.22) | 6.9 (3.2) ^A^ | 7.2 (3.3) ^A^ | 4.3 (1.9) ^B^ | <0,001 |
| GHGE per 2000 kcal | 5.38 (1.62) | 5.5 (1.6) ^A^ | 5.7 (1.6) ^A^ | 4.8 (1.5) ^B^ | <0,001 |
| **Organic and local scores** |  |  |  |  |  |
| Organic score (range [0;2]) | 0.5 (0.4) | 0.7 (0.5) ^A^ | 0.6 (0.4) ^B^ | 0.5 (0.4) ^B^ | 0,009 |
| Local score (range [0;2]) | 0.5 (0.4) | 0.6 (0.5) ^A^ | 0.6 (0.4) ^A,B^ | 0.5 (0.4) ^B^ | 0,054 |

Means with the same letter are not significantly different at alpha=0.05 level, after Bonferroni correction for multiple comparisons.

**S7. French university students’ characteristics across dietary groups after removing under and over reporters for energy intake (sensitivity analyses)**

|  | | |  |  |  |
| --- | --- | --- | --- | --- | --- |
|  | **Healthy diet group**  N=96 | | **Western diet group**  N=189 | **Frugal diet group**  N=113 | **p** |
| **Age**, *years*, mean (SD) [range] | | 21.7 (3.0) ^A^ | 20.9 (2.5) ^B^ | 20.5 (2.2) ^B^ | 0.003 |
| **Gender**, *female*, n (%) | | 48 (50) | 102 (54) | 70 (62) | 0.197 |
| **Scholarship status**, *scholarship*, n (%) | | 37 (39) | 70 (37) | 42 (37) | 0.967 |
| **Nationality**, French, n (%) | | 90 (94) | 176 (93) | 105 (93) | 0.970 |
| **Number of years of being a student (Post High-school)**, *years*, mean (SD) [range] | | 3.6 (2.2) | 3.3 (2.2) | 3.0 (2.1) | 0.107 |
| **Type of institution**^*^, n (%) | |  |  |  | 0.325 |
| University | | 59 (61) | 118 (62) | 61 (54) |  |
| Others | | 37 (39) | 71 (38) | 52 (46) |  |
| **Field of studies**^**^, n (%) | |  |  |  | 0.679 |
| Scientific | | 59 (61) | 123 (65) | 76 (67) |  |
| Others | | 37 (39) | 66 (35) | 37 (33) |  |
| **Highest educational qualification of parents**, n (%) | |  |  |  | 0.640 |
| < High-school +2 years diploma | | 31 (33) | 59 (32) | 36 (33) |  |
| High-school +2 years diploma | | 22 (24) | 39 (21) | 15 (14) |  |
| High-school +3 or +4 years diploma | | 14 (15) | 31 (17) | 22 (20) |  |
| ≥ High-school +5 years diploma | | 26 (28) | 53 (29) | 36 (33) |  |
| **Place of living**, n (%) | |  |  |  | 0.003 |
| Parents’ home | | 20 (21) | 40 (21) | 11 (10) |  |
| Student’s home or boarding school | | 17 (18) | 42 (23) | 35 (31) |  |
| Independent accommodation | | 59 (61) | 105 (56) | 67 (59) |  |
| **Living alone**, n (%) | |  |  |  | 0.665 |
| Yes | | 42 (44) | 97 (51) | 65 (57) |  |
| No, with parents | | 20 (21) | 40 (21) | 11 (10) |  |
| No, with a partner or a flat mate | | 34 (35) | 52 (28) | 37 (33) |  |
| **Level of physical activity**, n (%) | |  |  |  | <0.001 |
| Low | | 3 (3) | 35 (18) | 15 (13) |  |
| Moderate | | 45 (47) | 92 (49) | 67 (59) |  |
| High | | 48 (50) | 62 (33) | 31 (28) |  |
| **Dieting status**, none, n (%) | | 87 (91) | 173 (91) | 108 (96) | 0.322 |
| **Willingness to gain body mass**, yes, n (%) | | 27 (28) | 22 (12) | 11 (10) | <0.001 |
| **BMI**, **body-mass index**, *kg/m^2^*, mean (SD) | | 22.5 (3.3) | 22.4 (4.2) | 21.6 (3.5) |  |
| **WHO classification of weight status**, n (%) | |  |  |  | 0.264 |
| Underweight | | 5 (5) | 23 (12) | 14 (13) |  |
| Normal weight | | 74 (77) | 131 (69) | 84 (74) |  |
| Overweight or obese | | 17 (18) | 35 (19) | 15 (13) |  |
| **Declared diet**, n (%) | |  |  |  | 0.209 |
| Omnivore | | 64 (66.7) | 138 (73) | 72 (64) |  |
| Flexitarian, vegetarian or vegan | | 32 (33) | 51 (27) | 41 (36) |  |

^*^ Type of institution responses grouped together in “**others**”: “engineering school”, “business school”, “art school”, “higher school preparatory classes”, “technician school” and “others”

^**^ “Field of studies” responses grouped together in “**Science**”: “Industry”, “Health” and “Sciences” and “**Humanities**”: “art”, “business”, “law”, “teaching”, “humanities and languages”, “social sciences” and “political science”)

1. [↑](#footnote-ref-1)
